# Evaluating Oilseed Biofuel Production Feasibility in California’s San Joaquin Valley Using Geophysical and Remote Sensing Techniques

**DOI:** 10.3390/s17102343

**Published:** 2017-10-14

**Authors:** Dennis L. Corwin, Kevin Yemoto, Wes Clary, Gary Banuelos, Todd H. Skaggs, Scott M. Lesch, Elia Scudiero

**Affiliations:** 1 USDA-ARS, U.S. Salinity Laboratory, 450 West Big Springs Road, Riverside, CA 92507, USA; Todd.Skagggs@ars.usda.gov (T.H.S.); Elia.Scudiero@ars.usda.gov (E.S.); 2 USDA-ARS, Water Management Systems Research, 2150 Center Ave., NRRC Building D, Fort Collins, CO 80526, USA; Kevin.Yemoto@ars.usda.gov; 3 Department of Earth and Planetary Sciences, Northrop Hall, 221 Yale Blvd. NE, University of New Mexico, Albuquerque, NM 87131, USA; wesclary@unm.edu; 4 USDA-ARS, Water Management Research Unit, San Joaquin Valley Agricultural Sciences Center, 9611 South Riverbend Ave., Parlier, CA 93648, USA; Gary.Banuelos@ars.usda.gov; 5 Riverside Public Utilities—Resources Division, 3435 14th St., Riverside, CA 92501, USA; SLesch@riversideca.gov

**Keywords:** apparent soil electrical conductivity, EC_a_-directed soil sampling, electromagnetic induction, proximal sensor, response surface sampling, salt tolerance, boron tolerance, soil mapping, soil salinity, spatial variability

## Abstract

Though more costly than petroleum-based fuels and a minor component of overall military fuel sources, biofuels are nonetheless strategically valuable to the military because of intentional reliance on multiple, reliable, secure fuel sources. Significant reduction in oilseed biofuel cost occurs when grown on marginally productive saline-sodic soils plentiful in California’s San Joaquin Valley (SJV). The objective is to evaluate the feasibility of oilseed production on marginal soils in the SJV to support a 115 ML yr^−1^ biofuel conversion facility. The feasibility evaluation involves: (1) development of an Ida Gold mustard oilseed yield model for marginal soils; (2) identification of marginally productive soils; (3) development of a spatial database of edaphic factors influencing oilseed yield and (4) performance of Monte Carlo simulations showing potential biofuel production on marginally productive SJV soils. The model indicates oilseed yield is related to boron, salinity, leaching fraction, and water content at field capacity. Monte Carlo simulations for the entire SJV fit a shifted gamma probability density function: Q = 68.986 + gamma (6.134,5.285), where Q is biofuel production in ML yr^−1^. The shifted gamma cumulative density function indicates a 0.15–0.17 probability of meeting the target biofuel-production level of 115 ML yr^−1^, making adequate biofuel production unlikely.

## 1. Introduction

The United States is the world’s largest consumer of crude oil, resulting in two major problems: (1) low energy security and (2) high greenhouse gas (GHG) emissions. To increase national energy independence and decrease GHG emissions, the U.S. Congress enacted the Energy Independence and Security Act (EISA) in 2007 (110. P.L. 140). The EISA aims to increase the production of clean renewable fuels within the USA. Biofuels, such as biodiesel and renewable jet fuel from oilseed crops, are an alternative to petroleum-based fuels [1]. As part of EISA, the Renewable Fuel Standard (RFS) mandates the production of 137 billion liters of biofuel annually by 2022.

### 1.1. U.S. Military’s Need for Alternative Fuels

The Department of Defense is the leading consumer of fuel within the USA. The U.S. military consumes 23 billion liters of aviation fuel a year. The United States military desires a secure fuel source that is not threatened or controlled by world events. Biofuels developed from crops grown within the borders of the USA are a secure source of fuel uninfluenced by the same world events that affect petroleum-based fuels. To diversify their fuel sources the U.S. military set a goal of 5% of their yearly aviation fuel needs (1.15 billion liters per year) from biofuel.

Even though alternative fuels may not be price competitive, there is a long-term commitment by the military to use diversification in fuel sources as a means of reducing risk [2]. The military’s reliance on alternative fuels is strategic to help to ensure their operational readiness by increasing the ability to use multiple reliable fuel sources, thereby reducing dependence on any single fuel source that would make military decisions vulnerable to foreign manipulation. Interest in biofuels stems (1) from their potential to improve U.S. energy security because they are from renewable domestic sources that are theoretically unlimited over time and (2) from their potential to reduce GHG emissions, which is largely dependent on how the biofuels are produced and what land-use or land-cover changes occur [3].

### 1.2. Need for a Feasibility Study of Biofuel Production on Marginal Land in the San Joaquin Valley

Technology is available to produce biofuels, such as biodiesel and renewable jet fuel, from oilseed crops. The common oilseed crops for temperate regions include canola (rapeseed), sunflower, soybean, flax, safflower, and mustard. Some oilseed crops, such as mustard, show considerable salt tolerance.

Because of its climate, which enables year-round crop growth, and its reasonably secure source of irrigation water from surface, ground, and/or degraded water sources, California’s San Joaquin Valley (SJV) is an ideal agricultural area for a secure source of biofuel. In the SJV, biofuel feedstock that can grow on marginally productive soil of poor quality, particularly saline soils, is advantageous for cost reduction since marginal soils are usually fallow or produce yields too low to be profitable. The U.S. Department of Agriculture and the U.S. Navy’s Office of Naval Research identified the SJV as a potentially strategic location for biofuel production to meet 10% of the yearly biofuel production of aviation fuel (115 ML yr^−1^).

Marginally productive lands in California are an ample potential resource that can be used to great advantage to reduce cost. In the early 1980s, Backlund and Hoppes [4] estimated that the entire SJV had approximately 8.9 × 10^5^ ha of marginally productive saline-sodic soil, much of which resided on the west side of the SJV (WSJV). Current estimates of salt-affected soil for the WSJV using National Resource Conservation Service’s (NRCS) Soil Survey Geographic Database (SSURGO; https://websoilsurvey.nrcs.usda.gov/) are 3.6 × 10^5^ ha. Recent estimates using satellite imagery place this even higher at 5.5 × 10^5^ ha [5].

Regardless of the estimate that is accepted, there is extensive salt-affected soil within the SJV to grow salt-tolerant oilseed crops for conversion to biofuel that would not compete with food crops for land use. Salt and drought tolerant biofuel feedstock, such as mustard oilseed, has tremendous potential when grown on marginally productive salt-affected soils. Specially bred mustard varieties, such as Ida Gold mustard (*Sinapis alba* L.), are salt tolerant and produce reasonably high oil yields. Moreover, after the oil is pressed out from its seeds, the residual Ida Gold mustard seed meal can act as an effective biodegradable bioherbicide and can provide Se as a nutritional supplement in livestock feed. Se-enriched seed meal is a unique and extra cash-value product that can only be produced in the Se-laden soils of the WSJV.

Legislative mandates and incentives, volatility in oil prices, and advances in research and technology are driving the expectations of major increases in biofuel production as a viable alternative fuel source. USDOE and USDA [6] recommend the need for greater research to identify viable biofuel feedstock production and management systems to support bio-refineries at commercially viable capacities, i.e., 115 ML yr^−1^ or more. However, it is unknown if sufficient oilseed production could support a biofuel conversion facility of sufficient capacity to be economically viable, i.e., cost less than $1 L^−1^. Before considering an in-depth analysis of the economic viability and commercialization of biofuel, answers to basic questions are needed. The most fundamental question is, can sufficient biofuel feedstock be grown to support a biofuel conversion facility within agricultural regions of the USA, whether the Southwest, Midwest, or Southeast? All are potential agricultural areas proposed for biofuel production. More specifically, can Ida Gold mustard oilseed grow with sufficient yields on marginally productive salt-affected soils in the SJV to support a 115 ML yr^−1^ conversion facility?

### 1.3. Objective

It is the objective of this study (1) to formulate a crop yield model relating Ida Gold mustard oilseed yield to edaphic properties for the WSJV and (2) to use the crop yield model to predict the yield of Ida Gold mustard oilseed on salt-affected soils (i.e., soils with an EC_e_ greater than 4 dS m^−1^) for evaluating the feasibility of oilseed production on marginal soils to support a 115 ML yr^−1^ biofuel conversion facility in the SJV.

## 2. Materials and Methods

**Figure 1:**
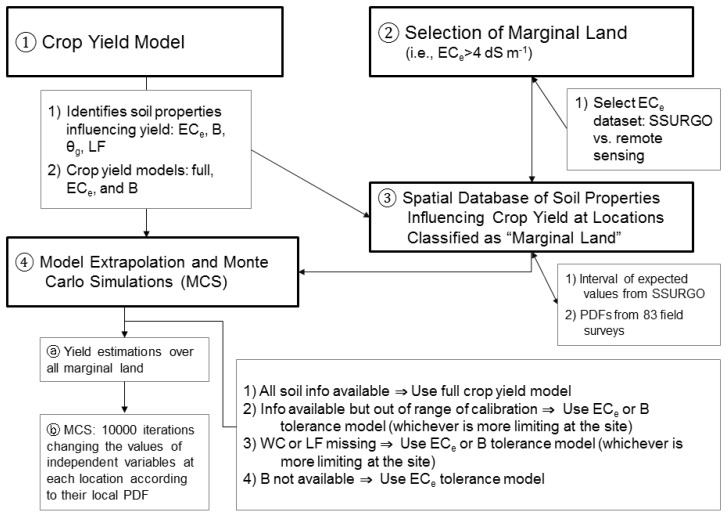
Flow chart of the feasibility evaluation of oilseed production on marginal soils in the San Joaquin Valley showing the four steps and flow of information.

The feasibility evaluation involves four steps: (1) development of an Ida Gold mustard oilseed yield model for marginal SJV soils using apparent soil electrical conductivity (EC_a_) directed soil sampling; (2) identification of marginally productive salt-affected soils for oilseed production in the SJV; (3) development of a spatial database of edaphic factors influencing mustard yield for the SJV derived from satellite imagery and the SSURGO database and (4) applying the Ida Gold mustard oilseed yield model on marginally productive soil for the SJV and performing Monte Carlo simulations to show the range and probability of potential biofuel production in the region. Figure 1 provides a flow chart showing the four steps and the flow of information.

### 2.1. Development of a Field-Based Ida Gold Mustard Oilseed Yield Model

A field experiment was conducted to identify the edaphic properties that influence oilseed yield of Ida Gold mustard. The approach of Corwin et al. [7] was followed. The approach uses geospatial electromagnetic induction measurements of EC_a_ to direct soil sampling for determining the soil properties influencing crop yield. The approach is based on the concept that if a correlation exists between crop yield and EC_a_, then EC_a_ is measuring, either directly or indirectly, one or more soil properties that are influencing the crop yield. By conducting an EC_a_ survey to direct soil sampling, crop yield/soil sampling sites can be identified that provide a range of edaphic properties and their influence on yield.

The development of a field-based Ida Gold mustard oilseed yield model involves the following steps: (1) selection of an appropriate field that has a full range of edaphic properties that are suspected of influencing Ida Gold mustard oilseed yield; (2) conducting an intensive EC_a_ survey of the field; (3) identification of sites within the field where crop yield and soil core samples are taken that reflect the range and variability of edaphic influences on oilseed yield; (4) analysis of chemical and physical properties of the soil cores thought to influence yield and (5) statistical analysis and crop yield model formulation.

#### 2.1.1. Study site description

**Figure 2:**
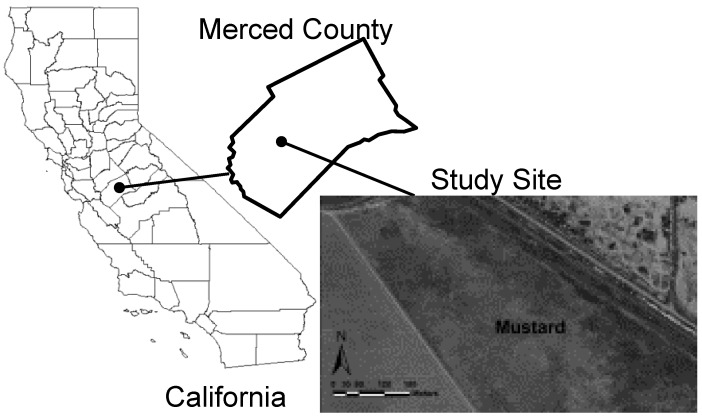
Map showing the location of the 16.2-ha field in California’s Merced County.

The study site was a 16.2-ha field (latitude-longitude coordinates: 37º02′02.97′′N, 120º47′31.56′′) located west of Los Banos in Merced County, California on the WSJV (Figure 2). The site provided a range of soil properties thought to influence the yield of Ida Gold mustard oilseed. In particular, the field was characterized by a broad range of salinity and boron values. Salinity and boron are properties common to marginally productive soils in the SJV that are known to significantly influence Ida Gold mustard oilseed yield. The soil at the study site is a Britto clay loam. The soil taxonomic class is fine, smectitic, thermic Typic Natraqualfs. The Britto series consists of deep, very poorly drained soils with high concentrations of salt and alkali in the lower horizons. The soil ranges from moderately saline to strongly saline (8–16 dS m^–1^). The texture in the top 55 cm is a clay loam and 0.55–1.55 m is a sandy clay loam. The parent material is alluvium derived from sedimentary rock. The mean annual precipitation is 25 cm.

#### 2.1.2. Preliminary and Intensive Apparent Soil Electrical Conductivity Surveys

Preliminary and intensive EC_a_ surveys were conducted on 14 January and 28 January 2014, following a pre-plant irrigation to bring the water content in the root zone to field capacity. The methods and materials used in the EC_a_ surveys followed the protocols and guidelines outlined in Corwin and Lesch [8,9,10]. An EM38 Dual Dipole electrical conductivity meter (Geonics Ltd., Mississaugua, Ontario, Canada. Product identification is provided for the benefit of the reader and does not imply endorsement by USDA.) connected to a GPS and mounted on a non-metallic sled was used in the EC_a_ surveys. Geospatial EC_a_ measurements in the vertical (EM_v_) and horizontal (EM_h_) coil configurations were taken simultaneously every 3–5 m. Each EC_a_ measurement was geo-referenced using GPS. The GPS receiver accuracy had sub-meter accuracy. The preliminary EC_a_ survey determined whether the study site provided a sufficiently wide range in soil properties influencing Ida Gold mustard oilseed yield to meet the objective of formulating a crop yield model. The preliminary survey consisted of making six east-west traverses. From the geospatial EC_a_ data set, six locations were selected to take core samples (0–1.5 m depth increment), which were immediately analyzed for pH_e_, saturation percentage (SP), B, and EC_e_.

**Figure 3:**
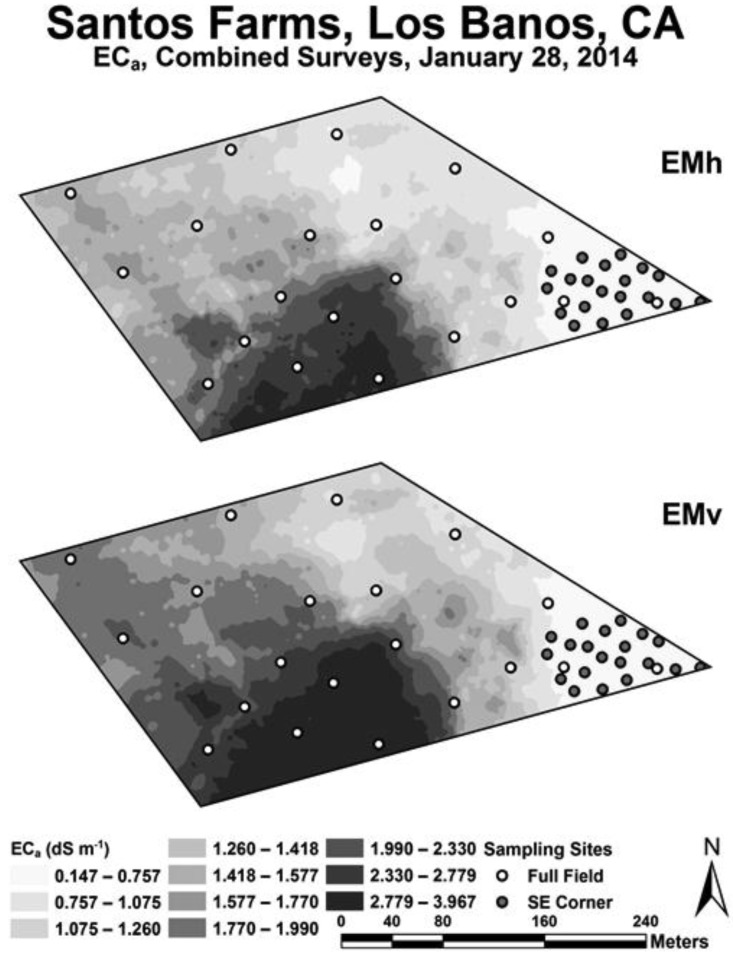
Maps of the apparent soil electrical conductivity (EC_a_) surveys taken with electromagnetic induction in the horizontal (EM_h_) and vertical (EM_v_) coil configurations, combining entire (full) 16.2-ha field and southeast (SE) corner surveys. Soil core sample sites are indicated by the clear and filled-in circles. The clear circles are soil sample sites selected from the full field EC_a_ survey and the filled-in circles are from the SE corner EC_a_ survey.

Following the preliminary EC_a_ survey, two separate intensive EC_a_ surveys were conducted. One EC_a_ survey for the entire 16.2 ha and another survey confined to the southeastern corner of the field. Figure 3 shows maps of a composite of the EC_a_ survey data for EM_v_ and EM_h_. The reason for taking the two intensive EC_a_ surveys was because the preliminary cursory EC_a_ survey indicated the greatest variability in salinity over the range that would affect Ida Gold mustard oilseed yield was in the southeast corner of the field; consequently, a separate survey and crop yield/soil sampling were performed for the southeast corner. The intention was to provide a range of soil properties, particularly with regard to salinity and boron, which would influence Ida Gold mustard oilseed yield to varying degrees thereby providing the data to formulate a more robust statistical model of crop yield.

#### 2.1.3. Soil and Ida Gold Mustard Oilseed Yield Sampling Design

Once the two intensive EC_a_ surveys were completed, ESAP software version 2.10R [11,12,13] was used to identify sites where crop yield and soil core samples were taken based on the spatial variation in the EC_a_ survey data. The ESAP software package uses a model-based sampling strategy (i.e., response surface sampling design) to identify sample site locations. The ESAP software identifies sites that characterize the range and variation in the geospatial EC_a_ measurements, reflecting the observed spatial variability in EC_a_, while minimizing any clustering of the sample sites by maximizing the spatial uniformity of the sampling design across the study area. A detailed discussion of the application of the response surface sampling design using EC_a_ survey data is in Lesch et al. [12].

The sample design for the 16.2-ha field consisted of 20 sample site locations for the entire field and 20 sample site locations for the southeast corner as selected by ESAP (Figure 3). Soil cores were taken at the 40 sites with a Giddings rig at six depth increments: 0–0.15, 0.15–0.30, 0.30–0.60, 0.60–0.90, 0.90–1.20, and 1.20–1.50 m. Duplicate soil samples were taken at eight sample site locations within 1 m of the original core to establish local-scale variability as explained in Corwin and Scudiero [14]. All soil samples were bagged in zip-lock bags and stored in an ice chest until refrigerated. A total of 288 soil samples were taken (6 depths at each site, 40 sites, and 8 duplicate sites). The depth to the water table was recorded as <1.5 m or >1.5 m.

At each of the 40 sample-site locations biomass and Ida Gold mustard oilseed yield were determined by hand within a 1 m^2^ area where each soil sample location was the centroid of the 1 m^2^ plant sample area. The biomass and oilseed yield were collected on 28–29 May 2014. Six of the 40 sample site locations had no Ida Gold mustard oilseed yield. All subsequent referral to yield is with respect to oilseed yield.

#### 2.1.4. Soil Chemical and Physical Analyses

In the field, a subsample (100–300 g) of each soil core sample was taken for soil moisture determination. The subsamples were weighed in the field to minimize error due to moisture evaporation. The subsamples were subsequently oven-dried at 110ºC for 24 h and weighed again to determine θ_g_. Saturation pastes were prepared for all 288 soil samples and saturation paste extracts were obtained following the procedure of Rhoades [15]. The saturation extracts were analyzed for the following properties: EC_e_, SP, pH_e_, 5 major anions (Cl^−^, HCO_3_^−^, PO_4_^3−^, NO_3_^−^, SO_4_^2−^), 4 major cations (Na^+^, K^+^, Ca^2+^, Mg^2+^), B, and sodium adsorption ratio (SAR). The chemical analysis procedures followed were those found in Sparks [16]. Leaching fraction (LF), defined as the ratio of the quantity of water draining past the root zone to that infiltrated into the soil’s surface, was estimated using two techniques: (1) the ratio of the EM_h_ EC_a_ divided by EM_v_ EC_a_ and (2) the ratio of Cl concentration in the irrigation water and Cl concentration at 1.2–1.5 m. The LF reflects the excess water applied to translocate salts from the root zone. Each property selected for analysis had the potential to influence Ida Gold mustard oilseed yield.

#### 2.1.5. Statistical Analysis and Ida Gold Oilseed Yield Model Formulations

Simple correlations were determined between yield and the edaphic properties of θ_g_, EC_e_, SP, pH_e_, Cl^−^, HCO_3_^−^, PO_4_^3−^, NO_3_^−^, SO_4_^2−^, Na^+^, K^+^, Ca^2+^, Mg^2+^, B, SAR, and LF. Correlations between the edaphic properties and EC_a_ and between oilseed yield and EC_a_ were determined. Scatter plots of yield vs. individual soil-related properties were also obtained.

The correlation analyses and scatter plots served as a basis for the development of the yield model for Ida Gold mustard oilseed. The correlations were useful to determine what properties were likely significant in influencing oilseed yield, while the scatter plots helped to determine the general form of the oilseed yield model. The oilseed yield model was developed using (spatial) multiple regression techniques [17]. The edaphic properties were the regressor or independent variables, and yield was the response or dependent variable. Backward variable selection was used to screen out the clearly nonsignificant edaphic properties with t-score values below 1.8. This predictor screening helped to filter out any multicollinearity in the regressor variables. Statistical data analyses were performed on the individual depth increment data (i.e., 0–0.15, 0.15–0.3, 0.3–0.6, 0.6–0.9, 0.9–1.2, and 1.2–1.5 m) and composite depth increment data (i.e., 0–0.15, 0–0.3, 0–0.6, 0–0.9, 0–1.2, and 0–1.5 m). Multiple regression modeling was performed on the composite depth increment data, and the increment characterized by the best goodness-of-fit was retained for further analyses.

In some instances, sufficient input data for the Ida Gold mustard oilseed yield model did not exist or fell outside the range of data that were used to develop the oilseed yield model. In those instances, the two-piece linear salt tolerance model of Maas and Hoffman [18] was used. For soil salinities exceeding the threshold salinity level, relative yield is estimated by the following equation:

(1) $$Y_{r}\;=\;100\;-\;b\;\left(\;\bar{EC_{e}}\;-\;a\right)$$

where *Y_r_* is the relative crop yield, *a* is the salinity threshold (dS m^−1^), *b* is the slope expressed in yield decrement percentage per dS m^−1^, and $\bar{EC_{e}}$ is the mean electrical conductivity of the saturation extract for the root zone (dS m^−1^). Maas and Hoffman [18] proposed that crop salt tolerance was represented by two linear lines, one a tolerance plateau with a slope of zero and the other shown in Equation (1) as a salinity dependent line whose slope was the yield reduction per unit increase in salinity. The point where both lines intersect is the salinity threshold, which represents the maximum soil salinity that does not reduce yield. The parameters *a* and *b* were determined from a compilation of salt tolerance data available for Ida Gold mustard oilseed for the SJV, including the data collected within this study and the work of Maas [19] and Grieve et al. [20]. If salinity was not limiting yield, then the B level determined the oilseed yield when B data were available. The three-piece trace element tolerance model presented in Page et al. [21] and first suggested by Burton et al. [22] was used. The salt and B tolerance data to develop these models were obtained from the 40 soil core and oilseed yield sample sites identified from EC_a_-directed soil sampling and from 10 supplemental sites. The locations of the supplemental sites were from a transect covering a range of yields. Salt tolerance data were from those sites varying in oilseed yield where all soil properties were optimal except salinity, which varied over a wide range, while B tolerance data were from those sites where all soil properties were optimal except B, which varied over a wide range. If no salinity and B data were available, then all properties were considered optimal for yield.

### 2.2. Identification of Salt-Affected Soils for Oilseed Production in the SJV

To assess the potential production of Ida Gold oilseed in the SJV, the yield model was applied over all salt-affected soils in the valley. Bohn et al. [23] defines salt-affected soils as soils with a root-zone salinity (i.e., EC_e_) above 4 dS m^−1^. Above 4 dS m^−1^ very sensitive, sensitive, and moderately salt-sensitive crops will show yield decrements. From 4–8 dS m^−1^ the yields of many crops are restricted, from 8–16 dS m^−1^ only salt tolerant crops yield satisfactorily, and above 16 dS m^−1^ only a few very tolerant crops produce satisfactory yields [24].

One means of identifying salt-affected soils in the SJV is the use of SSURGO. However, the accuracy and reliability of soil salinity in SSURGO is dubious because salinity is a spatially and temporally variable soil property influenced by crop and irrigation management strategies. Recent NRCS reports (e.g., [25]), provided salinity estimations only for non-irrigated farmland because the influence of irrigation on soil salinity cannot be accounted for using traditional soil survey protocols. Therefore, an evaluation of SSURGO’s accuracy with respect to soil salinity is needed. To evaluate the accuracy of SSURGO, salinity assessment surveys were performed on 22 agricultural fields (total area: 542 ha) scattered throughout the WSJV. In 2013, intensive EC_a_ surveys conducted at the 22 fields, collected 41,779 EC_a_ readings at an average density of 175 readings ha^−1^. Simultaneous EM_h_ and EM_v_ measurements of EC_a_ were taken. Across the 22 fields, 267 soil-sampling locations were identified using ESAP. Soil cores were taken to a depth of 1.2 m, representing the root-zone depth. Details of the EC_a_ survey and soil sampling are in Scudiero et al. [26].

Soil samples were analyzed for salinity (EC_e_; dS m^−1^), gravimetric water content (θ_g_; g g^−1^), pH_e_, and saturation percentage (SP) using procedures presented in *Methods of Soil Analysis Part 3* [16] and *Part 4* [27]. The SSURGO salinity data for the 22 fields were compared to both root-zone EC_e_ hard and EC_a_ soft data. The hard data (i.e., laboratory measurements of EC_e_ of the 0–1.2 m soil samples) were averaged for each field. The soft data (i.e., geospatial measurements of EC_a_) were calibrated to the EC_e_ data using spatial linear regression models [17] with an overall R^2^ = 0.93 by Scudiero et al. [26].

A map of EC_e_ using the soft data and EC_e_-EC_a_ calibration prepared for each field established spatial patterns for comparison to SSURGO map units. If the SSURGO database proved unreliable, then the regional-scale salinity assessment approach developed by Lobell et al. [28], which combines EC_a_-directed soil sampling with satellite imagery, would be used. This work has already been completed and published by Scudiero et al. [29] for the WSJV.

### 2.3. Development of a Spatial Database of Edaphic Properties Influencing Ida Gold Mustard Yield for the SJV

The most extensive spatial database of edaphic properties in the USA is the SSURGO Database. Collection of the information in SSURGO occurred by walking over the land and observing variation in the soil and vegetation to delineate map units. Characterization of soil properties within map units occurred by the collection of numerous soil samples and their analysis in the laboratory. Occasionally observation trenches are dug to characterize the horizonation. Soil maps outline areas called map units, which describe soils and other components that have unique properties, interpretations, and productivity. Collection of the information occurred at scales ranging from 1:12,000 to 1:63,360. The soils maps are intended for natural resource planning and management. Examples of information available from the database include available water capacity, texture, pH, electrical conductivity, and frequency of flooding; yields for cropland, woodland, rangeland, and pastureland; and limitations affecting recreational development, building site development, and other engineering uses. SSURGO map data can be viewed in the Web Soil Survey or downloaded in ESRI^®^ Shapefile format. Attribute data can be downloaded in text format. For the marginal soils of the SJV, information for water capacity, texture, pH, and frequency of flooding; yields for cropland, woodland, rangeland, and pastureland; and limitations affecting recreational development, building development, and other engineering uses was obtained through SSURGO.

To supplement the SSURGO data an extensive spatial database of quantitative soils information (i.e., EC_e_, pH_e_, saturation percentage, B, available water content, LF) for the SJV exists. The supplemental data set, collected over a period of 25 years by Corwin and colleagues, is a compilation of data that appeared in publications by Bourgault et al. [30], Corwin [31], Corwin and Lesch [8,9,10,32], Corwin et al. [7,33,34,35,36], Lesch and Corwin [17], Lesch et al. [37,38,39], Loague et al. [40], Rhoades et al. [41], Sanden et al. [42], and Scudiero et al. [26,29,43]. The supplemental data set consisted of edaphic property data from 83 fields within the SJV ranging in spatial extent from 0.4 to 65 ha with from 6 to 72 sample sites within a field. Soil samples were collected at 0.3-m increments to a minimum depth of 1.2 m and occasionally to 1.5 and 1.8 m. The supplemental data were used to determine frequency distributions (i.e., histograms), averages, ranges, and standard deviations for those properties found to influence Ida Gold mustard oilseed yield in the SJV. From this statistical information probability density functions (PDFs) were developed for LF, θ_g_, and B for the composite depth increment of 0–1.2 m, which was found to be the root zone depth for Ida Gold mustard oilseed. In general, SSURGO provides water content and B ranges associated with soil type. The PDFs, defined within the ranges of water content and B provided by SSURGO, were used as input for Monte Carlo simulations with the oilseed yield model. In instances where ranges of water content or B were not given in SSURGO, then B was assumed optimal and water content was estimated from a pedotransfer function using SSURGO texture data.

The use of degraded soil is crucial to driving down the cost of biofuel production in the SJV. Subsequently, the reliability of spatial salinity data for the SJV was of paramount importance for identifying salt-affected soils and for model input data. There were concerns regarding the reliability of the salinity ranges provided in SSURGO for the root zone due to anthropogenic influences (e.g., leaching of salts due to irrigation); consequently, as discussed in detail in Section 2.2 an evaluation of the reliability of SSURGO root-zone salinity was conducted by comparison to salinity ground-truth measurements of 22 fields in the WSJV presented by Scudiero et al. [26]. If SSURGO salinity in the root zone proved unreliable, then salinity predictions from the regional-scale soil salinity model of Scudiero et al. [29] were used. Scudiero et al. [29] found that as salinity increased, the prediction error increased, and quantified the error within salinity categories (i.e., 0–2, 2–4, 4–8, 8–16, >16 dS m^−1^). To incorporate this uncertainty into the Monte Carlo simulations, PDFs were established for each salinity category. The PDFs were defined by the average residuals and standard deviation of the residuals between observed salinities from the ground-truth salinity measurements of Scudiero et al. [26] and predicted salinities from the regional-scale soil salinity model of Scudiero et al. [29].

Leaching fraction is a difficult edaphic property to obtain and is not found in SSURGO. The supplemental data set of Corwin and colleagues was used to determine the frequency distribution, average, range, and standard deviation of LF for the SJV. Leaching fraction was determined from a ratio of EM_h_ EC_a_ to EM_v_ EC_a_ and from a ratio of Cl in irrigation water to Cl concentration below the root zone). Only those LFs where EM and Cl ratios agreed to within 5% were used.

### 2.4. Feasibility of Biofuel Production for the SJV: Application of the Yield Model and Monte Carlo Simulations

Monte Carlo simulations with the Ida Gold mustard oilseed yield model were performed with 10,000 iterations to provide a range of potential yields (kg ha^−1^) and probability of those yields, which are easily converted to L ha^−1^ of biofuel. This was done first for the WSJV and then for the entire SJV. The mean, median, standard deviation, skewness, and kurtosis of the Monte Carlo simulation distribution were calculated to characterize quantitatively the PDF and subsequently derive the cumulative density function (CDF) of biofuel production. Once the CDF is known, the probability (and thereby feasibility) of oilseed production in the SJV to support sufficiently a conversion facility becomes evident.

Sufficient input data was not always available for each field in the WSJV or SJV or sometimes the input data was outside the range of data used to develop the crop yield model for Ida Gold mustard oilseed. For these instances, an alternative model was needed (see Figure 1). When a complete set of input data was available at a field location, then the full crop yield model was used. For instances where input data was available but was outside the range of data from which the crop yield model was formulated, then either the EC_e_ (i.e., Equation (1)) or B tolerance model was used, whichever was more limiting at the site. For instances where insufficient input data existed and only EC_e_ and B input data were available, then either the EC_e_ (i.e., Equation (1)) or B tolerance model was used, whichever was more limiting at the site. If insufficient input data existed and only EC_e_ input data was available, then the EC_e_ tolerance model (Equation (1)) was used.

## 3. Results and Discussion

The preliminary EC_a_ survey revealed that the greatest variation in salinity over the range that would result in oilseed yield decrements was in the southeast corner of the study site; consequently, two intensive EC_a_ surveys were conducted with separate soil sampling designs for each survey. One EC_a_ survey and associated soil sampling covered the full field and the other focused on the SE corner. Figure 3 shows maps of the horizontal coil configuration (EM_h_) and vertical coil configuration (EM_v_) EC_a_ surveys. The combined soil sampling designs (i.e., full field and southeast corner) provided a full range of soil properties and oilseed yields to build a robust Ida Gold mustard oilseed yield model. The only property potentially influencing Ida Gold mustard oilseed yield that did not vary significantly at the study site was texture. In general, the fine-textured soils (mainly loams and clay loams), which predominate the WSJV, do not vary to a significant extent because the soil is a consequence of lacustrine deposits and of fine-grained alluvium material originating from the Coastal Ranges [44,45].

**Figure 4:**
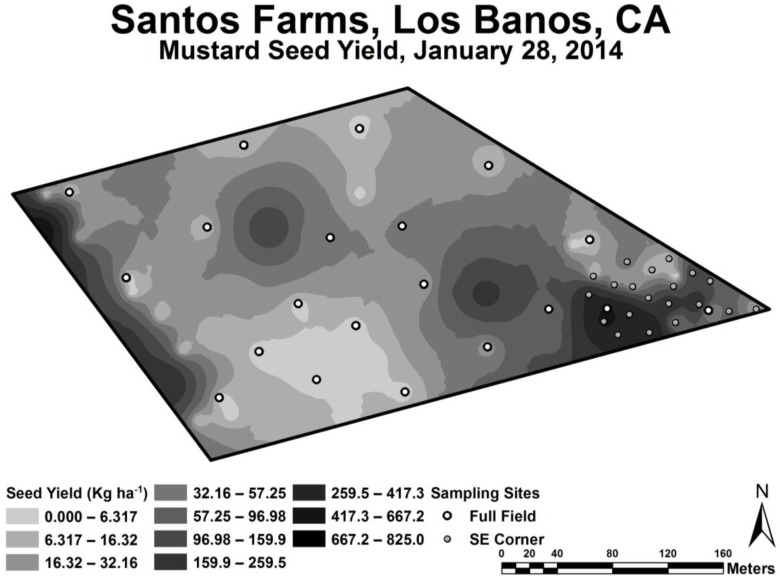
Map of Ida Gold mustard oilseed yield. Oilseed yield sample sites (1 m^2^ sample area) are indicated by the clear and filled-in circles. The clear circles are oilseed yield sample sites selected from the full field EC_a_ survey and the filled-in circles are from the SE corner EC_a_ survey.

Geospatial EC_a_ measurements, both EM_h_ and EM_v_, were higher on the west side of the 16.2-ha field than on the east side (see Figure 3). The lowest EC_a_ measurements were in the southeast corner, where EC_a_ ranged from 0.15 to 0.76 dS m^−1^. Over this range of EC_a_ the yield was found to vary the greatest (see Figure 4) and therefore provided the most useful data for oilseed yield model formulation. The range of EC_a_ over the entire 16.2 ha was 0.15 to 3.97 dS m^−1^. Oilseed yield over the EC_a_ range of 0.76 to 3.97 dS m^−1^ tended to be low. Yield significantly correlated to both EM_h_ and EM_v_ EC_a_, with correlation coefficients of 0.68 and 0.51, respectively. The higher correlation of EM_h_ EC_a_ to oilseed yield suggests that the root zone for Ida Gold mustard was around 1 m since the EM_h_ measurement penetrates to a depth of approximately 1 m, while EM_v_ penetrates to approximately 1.5 m. The fact that EC_a_ and oilseed yield correlated indicates that EC_a_ must be measuring a soil property or properties that influence oilseed yield; therefore, the response surface sampling design will successfully map the property or properties [9].

The measured edaphic properties that were felt to potentially influence Ida Gold mustard oilseed yield included: EC_e_, θ_g_, SP, pH_e_, trace elements (B, Se, As, Mo), major cations (Na^+^, K^+^, Ca^2+^, Mg^2+^), major anions (Cl^−^, HCO_3_^−^, PO_4_^3−^, NO_3_^−^, SO_4_^2−^), SAR, micro-elevation, depth to groundwater, and groundwater EC. Table 1 is a summary by depth of these edaphic properties, except for micro-elevation, depth to groundwater, and groundwater EC.

Table 1 reveals patterns in the field-wide soil profile. Field-wide average soil salinity (EC_e_) increases with depth up to the 0.3–0.6, levels off at the 0.3–0.6 and 0.6–0.9 m depth increments, and then decreases with depth. Saturation percentage (SP) is reasonably constant over depth ranging from means of 48.13% at 1.2–1.5 m to 54.85% at 0.3–0.6 m, indicating uniform texture through the soil profile. Gravimetric water content (θ_g_) at field capacity increases with depth from 0.10 kg kg^−1^ at 0–0.15 m to 0.24 kg kg^−1^ at 1.2–1.5 m. Boron levels tend to be lower below 0.3–0.6 m. pH increases with depth from 7.29 at 0–0.15 m to 7.98 at 1.2–1.5 m. SAR increases with depth from 6.53 at 0–0.15 m to 19.04 at 0.3–0.6 m, then decreases to 10.72 at 1.2–1.5 m.

Table 2a presents mean and range statistics, standard deviation, standard error, coefficient of variation, skewness, and kurtosis for the composite 0–1.5 m depth. The highest coefficients of variation (CVs) are for EC_e_, various cations and anions (e.g., Na^+^, Ca^2+^, SO_4_^2−^, PO_4_^−^, and Cl^−^), and SAR, while the lowest CVs are for pH_e_, SP, and θ_g_. All edaphic properties in Table 2a are positively skewed. Most properties show a positive kurtosis except θ_g_, SP, HCO_3_^−^, and B. The range, minimum, and maximum of the edaphic properties in Table 2a are of particular interest because they confirm that the study site is well suited for developing a crop yield model based on edaphic properties since a wide range of edaphic conditions influencing oilseed yield are present. For instance, EC_e_, pH_e_, B, and SAR cover broad ranges from low to very high. For the composite depth of 0–1.5 m, EC_e_ ranged from 2.05 to 36.22 dS m^−1^, pH_e_ ranged from 7.18 to 8.53, B ranged from 2.65 to 35.06 mg L^−1^, and SAR ranged from 3.48 to 112.13. The SP is the only soil property that is narrow in range, nonetheless it reflects a texture that is typical of the WSJV.

### 3.1. Ida Gold Mustard Oilseed Yield Models for Marginal SJV Soils

Exploratory statistical analyses revealed that Ida Gold mustard oilseed yield was most significantly correlated to individual edaphic properties for the top 1.2 m. Table 2b presents mean and range statistics, standard deviation, standard error, coefficient of variation, skewness, and kurtosis for the composite 0–1.2 m depth. Table 3 presents simple correlations between edaphic properties and both EC_a_ and oilseed yield for the 0–1.2 m depth increment. The edaphic properties most significantly correlated to EC_a_ include θ_g_, EC_e_, B, SAR, LF (determined by the ratio EM_h_ EC_a_ /EMv EC_a_), and SP. The edaphic properties most significantly correlated to oilseed yield include θ_g_, EC_e_, B, LF, and SP.

Corwin and Lesch [9] indicate that the depth increment or composite depth increment associated with the best-fitting yield model (i.e., highest R^2^) reflects the root zone of the crop. The top 1.2 m resulted in the most statistically significant and best-fit Ida Gold mustard oilseed yield model (see Equation (3)). Consequently, the 0–1.2 m soil interval was taken to represent the root zone of Ida Gold mustard at the study site. Subsequently, all data presented and discussed are with respect to the 0–1.2 m composite depth or to individual depth increments that lie within the composite depth of 0–1.2 m.

Exploratory statistical analyses revealed that θ_g_, EC_e_, B, and LF were the most influential edaphic properties on oilseed yield. Scatter plots of these properties vs oilseed yield indicated quadratic relationships of EC_e_ and B to oilseed yield and linear relationships of LF and θ_g_ to yield. Based on the initial exploratory correlation and multiple linear regression analysis, the following regression model structure was proposed to describe edaphic property effects on oilseed yield:

*Y* = *β_0_* + *β_1_*(*B*) + *β_2_*(*B*)^2^ + *β_3_*(*EC_e_*) + *β_4_*(*EC_e_*)^2^ + *β_5_*(*LF*) + *β_6_*(*θ_g_*) + *ε* (2)

where *Y* is the Ida Gold mustard oilseed yield (kg ha^−1^); *B* is boron concentration (mg L^−1^); *EC_e_* is electrical conductivity of the saturation extract (dS m^−1^); *LF* is the leaching fraction; *θ_g_* is the gravimetric water content (kg kg^−1^); *β_0_*, *β_1_*, *β_2_*, . . . , *β_6_* are the regression model parameters, and *ε* is the random error component, initially assumed to be normally distributed and spatially independent. Ordinary least squares (OLS) regression techniques resulted in a fitted regression equation with a R^2^ = 0.89 and adjusted R^2^ = 0.78. Adjusting for spatial autocorrelation using the maximum-likelihood approach resulted in the following Ida Gold mustard oilseed yield model (Equation (3)):

*Y* = 146.4(*B*) − 18.3(*B*)^2^ + 83.0(*EC_e_*) − 6.1(*EC_e_*)^2^ + 1301.0(*LF*) + 319.8(*θ_g_*) + 30.1 (3)

Equation (3) represents the most parsimonious and robust model for marginally productive salt-affected soils of the WSJV. Any locations where no oilseed yield was obtained were not used in the model development. The *LF* and *θ_g_* parameters are highly significant at or near the 0.01 level, and the *EC_e_* (linear and quadratic) and *B* (linear and quadratic) parameter estimates are significant at or near the 0.05 level. The *LF* and *θ_g_* parameters are both positive, implying that the yield increased as either *LF* or *θ_g_* increased, which is physically sound since increased leaching reduces osmotic stress and increased water content increases the plant-available water reducing matric stress. The positive linear and negative quadratic *EC_e_* terms imply that the yield increased at low *EC_e_* up to a point of maximum yield with respect to *EC_e_* and decreased beyond the maximum. The point of maximum yield with respect to *EC_e_* was calculated by setting the first partial derivative of the fitted regression to zero with respect to *EC_e_*, which resulted in a value of 6.8 dS m^−1^. Similarly, the positive linear and negative quadratic *B* terms imply that the yield increased under low *B* up to a point of maximum yield with respect to *B* and decreased beyond the maximum. The point of maximum yield with respect to *B* was 4 mg L^−1^.

**Figure 5:**
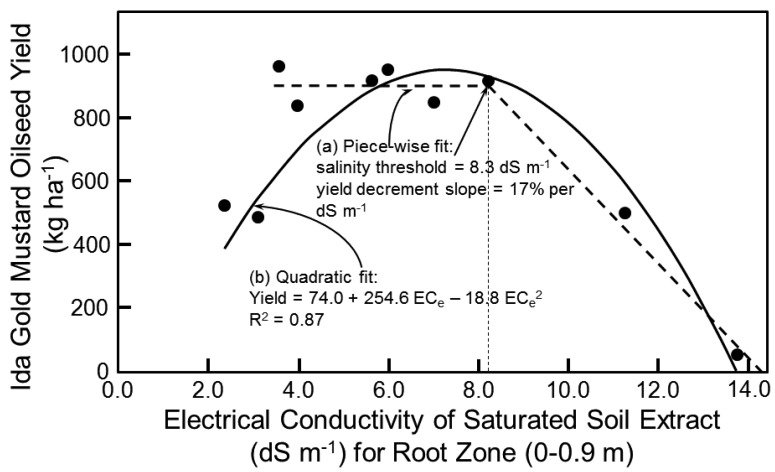
Salt tolerance models for Ida Gold mustard oilseed: (**a**) two-piece linear salt tolerance model (thick dashed line) of Maas and Hoffman [18] and (**b**) quadratic salt tolerance model (solid line). Thin dashed line indicates the salinity threshold of the two-piece linear salt tolerance model.

In those instances where sufficient input data for Equation (3) did not exist or fell outside the range of data that was used to develop the model, the two-piece linear salt tolerance model (i.e., Equation (1)) of Maas and Hoffman [18] was used to predict oilseed yield. Salt tolerance data at the study site established a salinity threshold of 8.3 dS m^−1^, which is the term *a* in Equation (1), and a yield decrement slope of 17%, which is the term *b* in Equation (1) (Figure 5a). The salinity threshold of 8.3 dS m^−1^ corresponds reasonably well with the salinity of maximum oilseed yield of 6.8 dS m^−1^ in Equation (3). Equation (1) established the upper limit of the salinity range of salt-affected soils that would grow Ida Gold mustard oilseed. A 17% yield decrement for each 1 dS m^−1^ increase in root-zone soil salinity beyond 8.3 dS m^−1^ resulted in no oilseed yield above 14.3 dS m^−1^. It is important to note that the salinity range of 4–14.3 dS m^−1^ was not necessarily economically viable. Once the feasibility of reaching the 115 ML yr^−1^ goal is established, then the maximum yield decrement that is economically viable could be determined. Viability would take into account other economically relevant factors such as (1) selling the seed meal remaining, after the oilseed has been pressed to extract the oil, as Se-enriched meal for livestock or as a herbicide used in organic agriculture and (2) incorporation of gasification to generate power to run the oil press.

The two-piece linear salt tolerance model (i.e., Equation (1)) of Maas and Hoffman [18] was not the best model to fit the data as seen in Figure 5a. A quadratic model (Figure 5b) actually fits the data best:

*Y* = 74.0 + 254.6 EC_e_ − 18.8 EC_e_^2^ (R^2^ = 0.87) (4)

By taking the derivative of Equation (4) with respect to EC_e_ and setting it equal to 0, EC_e_ corresponds to the peak mustard oilseed yield, which is 6.8 dS m^−1^. This is equal to the EC_e_ producing the maximum yield in Equation (3). Similarly, a quadratic relationship between yield and EC_e_ was also found by Corwin et al. [7] for cotton seed yield. The explanation for a quadratic relationship between EC_e_ and yield of cotton seed and mustard oilseed is that when the plant is osmotically stressed it puts energy into the development of the reproductive part of the plant rather than vegetative tissue; consequently, as EC_e_ increases up to the salinity threshold biomass yield of both crops remains constant or may decrease, while mustard oilseed and cotton seed yield steadily increase. After the salinity threshold is reached then oilseed and cotton seed yield and the biomass of both crops decrease as salinity increases. This quadratic relationship indicates that more breeding research is needed to obtain new cultivars of mustard that have their peaks of potential yield at different ranges of salinity to maximize production in the SJV.

**Figure 6:**
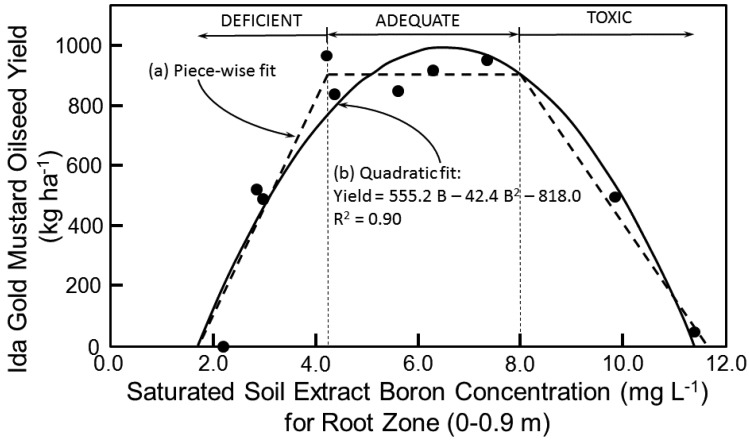
Boron tolerance models for Ida Gold mustard oilseed: (**a**) three-piece linear B tolerance model (thick dashed line) and (**b**) quadratic B tolerance model (solid line). Thin dashed lines indicate the B deficiency and toxicity thresholds.

Boron tolerance studies at the field site showed an excellent fit of the three-piece linear model with an optimum range for B of 4.2–8 mg L^−1^ (Figure 6a). Below 4.2 mg L^−1^ of B and above 8 mg L^−1^ the yield of Ida Gold mustard oilseed drops, where B < 4.2 mg L^−1^ is a B deficiency and B > 8 mg L^−1^ is a B toxicity. Above 8 mg L^−1^ the drop was 28% for every 1 mg L^−1^ increase in B with no yield occurring above 11.6 mg L^−1^ of B. A quadratic model (Equation (5)) produced a comparable fit to the B tolerance data (Figure 6b):

*Y* = 555.2B − 42.4B^2^ − 418.0 (R^2^ = 0.90) (5)

By taking the derivative of Equation (5) with respect to B and setting it equal to 0, B corresponds to the peak oilseed yield, which is 6.5 mg L^−1^.

The piece-wise linear models of Figure 5a and Figure 6a for salinity and B, respectively, are traditional models found throughout the literature. The piece-wise linear models were used in the Monte Carlo simulations. However, the quadratic models provided slightly better fits to the data; consequently, a second set of Monte Carlo simulations was performed with the salinity and B quadratic models, Equations (4) and (5), respectively.

### 3.2. Evaluation of SSURGO Soil Salinity Accuracy and Identification of Salt-Affected Soils

An evaluation of the accuracy of SSURGO spatial data for root-zone soil salinity revealed that only 5 out of 22 fields assessed the mean salinity or range in salinity accurately, suggesting that the more transient salinity levels and patterns in the root zone are not captured in the one-time measurements of NRCS soil surveys. However, SSURGO was able to assess 15 out of 22 fields accurately for salinity below the root zone, indicating that the salt levels below the root zone remained relatively unchanged and unaffected by anthropogenic influences. The failure of SSURGO to provide accurate root-zone soil salinity spatial data necessitated a reliance on the regional-scale salinity model developed by Scudiero et al. [29] as input data for the Ida Gold mustard oilseed yield model.

**Figure 7:**
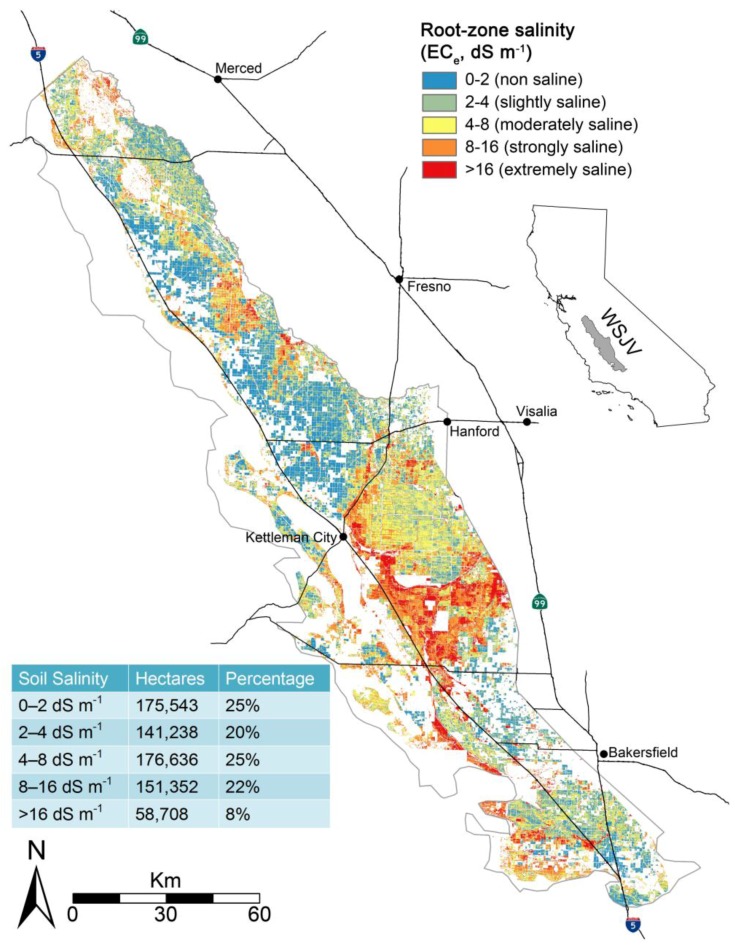
Map of salt-affected soils for the west side of California’s San Joaquin Valley (WSJV) estimated from the regional-scale soil salinity model of Scudiero et al. [5,29].

Because of the inaccuracy of salinity in SSURGO the Scudiero et al. [29] regional-scale salinity model was used to identify salt-affected soils (EC > 4 dS m^−1^) for the SJV. Figure 7 shows the extent of salt-affected soils for the WSJV as estimated by the Scudiero et al. [29] regional-scale salinity model. A comparison of total land cover greater than 4 dS m^−1^ for SSURGO and the Scudiero et al. [29] regional-scale salinity model indicates that SSURGO estimated 33% less salt-affected land for the WSJV. Furthermore, the distribution of salt-affected soils was concentrated in contiguous patterns along the eastern half of the WSJV for SSURGO, whereas the Scudiero et al. [29] salinity patterns were more diffusely spread as shown in Figure 7. Only fields identified as salt-affected were subsequently used in the Monte Carlo simulations. Any field where the field average root zone EC_e_ was estimated to be <4 dS m^−1^ was disregarded and not included in the Monte Carlo simulations.

### 3.3. Input Data for Ida Gold Mustard Oilseed Yield Model

The reliability and accuracy of the spatial data that serves as input into any model are just as critical as the model itself, exemplified by the old adage, garbage in garbage out. Sensitivity analysis of the crop yield model provides an indication of the input variables that need the greatest level of accuracy and therefore need particular scrutiny when building a spatial database of inputs for the crop yield model. Sensitivity analysis (Table 4) established the degree of influence that each edaphic property had on oilseed yield in Equation (3). The influence was determined by calculating how much the predicted oilseed yield changed when the value for each independent variable in Equation (3) was individually shifted by 1 standard deviation from its mean level or point of maximum yield with respect to the edaphic property. The means and standard deviations were obtained for the 0–1.2 m depth increment excluding any points where no yield occurred (Table 2b). A baseline yield was used as the point of reference to establish the percentage of change. A baseline value of 6.8 dS m^−1^ was used for salinity, rather than the mean EC_e_ level of 10.0 dS m^−1^ from Table 2b because the value 6.8 dS m^−1^ represents the point of maximum yield with respect to the quadratic salinity response pattern. For the same reason a B baseline of 4.0 mg L^−1^ was used in the sensitivity analysis. The calculated percentage yield change shown in Table 4 indicates that B is the most significant factor influencing Ida Gold mustard oilseed yield, followed by EC_e_, then LF, and finally θ_g_.

In the case of EC_e_, oilseed yield model input values were obtained from the 30 x 30 m predictions from Scudiero et al. [29] and used to determine the average EC_e_ for each field for the root zone (0–1.2 m) within the SJV. For the Monte Carlo simulations, PDFs were defined by the average residual and standard deviation of the residuals for each category of salinity (i.e., 0–1, 1–2, 2–3, 3–4, 4–5, 5–6, 6–7, 7–8, 8–9, 9–10, 10–11, 11–12, 12–13, 13–14, 14–15, 15–16, and >16 dS m^−1^) using the predicted and ground-truth EC_e_ data from Scudiero et al. [5,29]. The input EC_e_ of each pixel in the Monte Carlo simulations was determined from the predicted EC_e_ and residual PDF. Table 5 is a summary of the average residuals, standard deviation of the residuals, and data count for each salinity category and for the entire data set. All PDFs were normally distributed. The PDFs for categories 0–1, 1–2, 2–3, and 3–4 d S m^−1^ were not used since only those fields with an average root zone EC_e_ of greater than 4 dS m were used in the Monte Carlo simulations. There was not a substantial difference in the Monte Carlo simulation findings using the PDF for the entire data set in Table 5 as compared to the individual salinity categories; consequently, all subsequent Monte Carlo simulation discussions will be for the use of the PDF for the entire EC_e_ residual data set.

The edaphic property data collected and presented by Corwin and colleagues (Bourgault et al., [30]; Corwin [31]; Corwin and Lesch [8,9,10,32,46]; Corwin et al. [7,33,34,35,36]; Lesch and Corwin [17]; Lesch et al. [37,38,39]; Loague et al. [40]; Rhoades et al. [41]; Sanden et al. [42]; and Scudiero et al. [26,29,43]) over 2.5 decades of salinity assessment field studies were used to develop the PDFs for B, LF, and θ_g_ for the SJV, which were subsequently used as input data in the Monte Carlo simulations. The PDFs for B, LF, and θ_g_ were log normally distributed.

Oilseed yield must be converted to biofuel yield. The oil content of Ida Gold mustard oilseed was 26% and the extraction efficiency was 64%. Subsequently, for every 1000 kg ha^−1^ of oilseed produced then 166.4 kg ha^−1^ of 100% biofuel resulted, which represented 175.3 L ha^−1^ of biofuel. It is of importance to note that transesterified biofuel is generally blended with diesel (1:5 ratio of biofuel to diesel) and sold as 20% biofuel in California.

### 3.4. Monte Carlo Simulations Feasibility of Biofuel Production for the SJV

**Figure 8:**
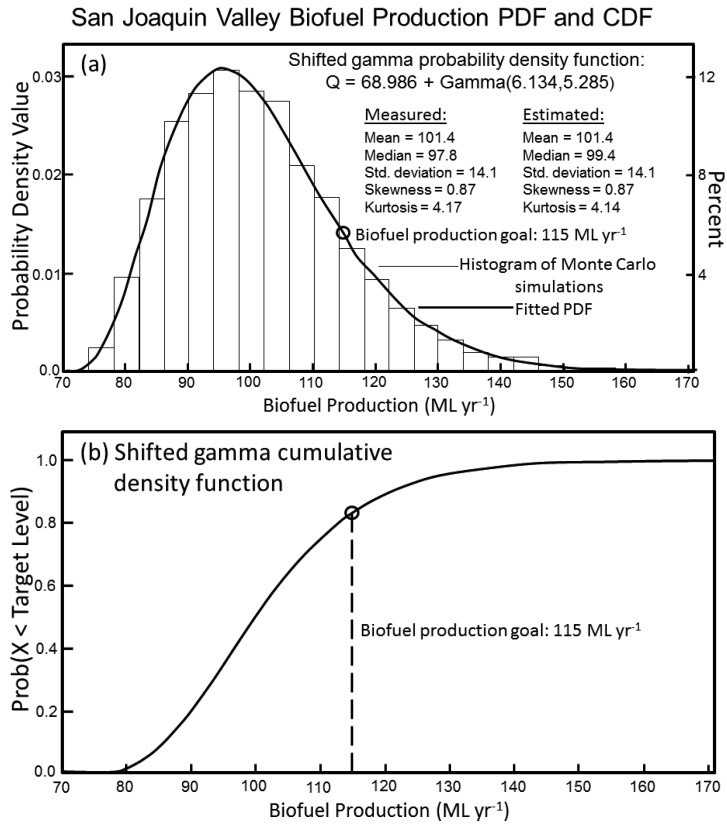
Biofuel production (**a**) histogram of Monte Carlo simulations and associated probability density function (PDF) and (**b**) cumulative density function (CDF) for Ida Gold mustard oilseed from San Joaquin Valley salt-affected soils (i.e., soils with salinity of > 4 dS m^−1^) based on 10,000 Monte Carlo simulations.

Monte Carlo simulations for the WSJV never produced a simulation that resulted in more than 71.9 ML yr^−1^ of biofuel produced, which is well below the goal of 115 ML yr^−1^. Subsequently, Monte Carlo simulations were performed for the entire SJV. To do this, the regional-scale salinity model of Scudiero et al. [29] was applied to the entire SJV to identify salt-affected soils, which totaled 9.7 × 10^5^ ha. Monte Carlo simulations resulted in the PDF and associated CDF shown in Figure 8a,b, respectively. The PDF and CDF are based on Monte Carlo simulations that incorporate the piece-wise models of salinity and B tolerance. The histogram of Monte Carlo simulations is best fit with the shifted gamma PDF in Equation (6) (Figure 8a):

Q = 68.986 + gamma(6.134,5.285)(6)

where Q is the biofuel production in ML yr^−1^. A comparison of the means, medians, standard deviations, skewness, and kurtosis for the measured Monte Carlo simulation data and for the estimates from Equation (6) shows excellent agreement, with means of 101.4 and 101.4 ML yr^−1^, medians of 97.8 and 99.4 ML yr^−1^, standard deviations of 14.1 and 14.1 ML yr^−1^, skewness of 0.87 and 0.87, and kurtosis of 4.17 and 4.14, respectively (Figure 8a). From the CDF (Figure 8b) there is a 17% probability of meeting the minimum production level when all salt-affected soils in the SJV are utilized for oilseed production. When the Monte Carlo simulations include quadratic salt and B tolerance models (i.e., Equations (5) and (6), respectively), there is little difference, with a 15% probability.

Several potential weaknesses in the approach need discussion as well as how the impacts of these weaknesses were mitigated in the Monte Carlo simulations. First, the oilseed yield model was developed from a single field with a fine-texture soil that did not vary significantly. Even though the study site may have been representative of many of the fine-textured soils of the WSJV, it was not representative of coarse-textured soils found in the east side of the SJV; consequently, the use of the oilseed yield model for the entire SJV is dubious. Second, the regional-scale salinity model of Scudiero et al. [29] was developed from a database that did not include tree crops or vineyards, which makes the input salinities for the oilseed yield model dubious for areas containing vineyards or tree crops. Scudiero et al. [5] showed that the regional-scale salinity model of Scudiero et al. [29] over estimates salinity levels for orchards and vineyards, which would render reduced oilseed yield estimates of Ida Gold mustard when planted between tree rows or would identify these lands as salt-affected when they are not. Third, applying the oilseed yield model outside the range of data that was used to develop it is problematic.

To rectify these problems, several precautions were taken. No areas where orchards or vineyards occurred were included in the Monte Carlo simulations. In fields where the texture was coarse, the oilseed yield model of Equation (3) was not applied. Rather, the two-piece linear salt tolerance model of Maas and Hoffman [18] presented in Equation (1) or the three-piece linear B tolerance model presented in Figure 6 were used, depending on which was most limiting to oilseed yield. Similarly, in cases where the input data for the oilseed yield model (Equation (3)) fell outside the range of data used to develop the model, then the two-piece linear salt tolerance model of Maas and Hoffman [18] or the three-piece linear B tolerance model were used instead.

Even though the feasibility of meeting desired oilseed production levels is dubious, there are circumstances that could improve the likelihood of meeting the 115 ML lower limit of production. Water in terms of water content and water available for leaching are two impactful properties pertaining to oilseed yield in the model. Maintaining the root zone water content at high levels with adequate leaching could drive up yields sufficiently high to make oilseed a viable biofuel in the SJV. In particular, high frequency irrigation to maintain root zone water contents near field capacity would have significant impact. However, this is only feasible when the SJV has sufficient irrigation water supplies, which may be the exception rather than the rule as shown by the recent 6-year California drought (2011–2016).

Orchards, whether in salt-affected (EC_e_ > 4 dS m^−1^) or non-salt-affected (EC_e_ ≤ 4 dS m^−1^) soils, could play a major role in enhancing the feasibility of biofuel production in the SJV. Planting mustard oilseed between rows in orchards, sometimes referred to as intercropping or alley cropping, provides tremendous acreage for crop growth on lands that are otherwise left fallow in many instances. Intercropping has several additional advantages, including weed control, wind and water erosion control, dust management, improved percolation, more effective use of land and resources, and additional crop revenue. There are roughly 130,000 ha of orchards in the SJV. By planting an oilseed crop between rows as a secondary crop, biofuel production could increase roughly 15–30%, which would increase the probability of meeting the 115 ML yr^−1^ target from 15–17% to 60–80% for the entire SJV. Even though intercropping with oilseed may make biofuel production feasible, it certainly does not necessarily make it economically viable. Furthermore, secondary crops from intercropping are a management challenge for producers filled with additional management problems.

Aside from meeting the 115 ML yr^−1^ target, economic viability is a concern. Growing oilseed on marginally productive soil is not the only means of lowering cost. Another resource that can lower biofuel production cost is the reuse of degraded water for irrigation. Corwin [31] showed in a long-term (12 years) study that the reuse of 3–5 dS m^−1^ drainage water was a viable means of reclaiming saline-sodic soil on the WSJV, supporting a salt-tolerant crop during the reclamation process, and providing financial return to the producer on otherwise non-productive farmland. In addition, drainage water is a particularly valuable alternative water resource during drought years in the SJV, provided drainage water is in sufficient supply.

Even though biofuel feasibility evaluation is a valuable application of sensor technology, the unique aspect of this study is that it presents an innovative regional-scale approach for modeling the interaction of edaphic properties on crop yield by characterizing the spatial variability of soil properties influencing yield using proximal and satellite sensors. In essence, each sampling location identified from an EC_a_ survey serves as an independent crop yield study looking at the interaction of edaphic properties on yield, thereby rendering a more robust model with lower long-term labor requirements than plant salt (or B) tolerance studies provided in the past. Furthermore, past plant salt tolerance studies did not evaluate the interaction of edaphic properties on crop yield.

The same approach can be used to predict drought impacts on crop productivity within an agricultural region, to identify reclamation needs to optimize crop productivity, and to manage resources (e.g., irrigation water, land, crop selection) and edaphic properties (e.g., salinity, B, etc.) to optimize crop yield. Because of its regional-scale application and quantitative probability assessment, the approach provides land and water resource specialists with a tool to make regional- and landscape-scale resource decisions with a clear understanding of the likelihood of impact on agricultural productivity.

## 4. Conclusions

This study exemplifies the combined use of proximal and satellite sensors to answer a practical agricultural question of national significance with strategic military implications concerning the feasibility of biofuel production in California’s San Joaquin Valley to help meet the aviation fuel needs of the U.S. military. A quantitative assessment of the probability of attaining a minimum of 115 ML yr^−1^ of biofuel from oilseed indicates that there is a 15–17% probability of meeting the minimum production level when all salt-affected soils in the SJV are utilized for oilseed production (Figure 8). The low probability of meeting the minimum production level and the fact that all salt-affected soils throughout the SJV, not just WSJV salt-affected soils, would need to be cropped with Ida Gold mustard to produce sufficient oilseed biofuel to support a 115 ML conversion plants makes the idea untenable. Furthermore, a significant portion of the 115 ML of biofuel would be produced on soils of moderate salinity (i.e., 4–6 dS m^−1^), moderate B levels (i.e., 4–8 mg L^−1^), high LF, and high available water content, which are soils that can either be easily reclaimed to produce higher cash crops or used to grow salt and B tolerant crops of higher cash return than oilseed, such as pistachio.

## Figures and Tables

**Table 1:** Mean and range statistics of soil edaphic properties for the depths within the root zone of 0–0.15, 0.15–0.3, 0.3–0.6, 0.6–0.9, 0.9–1.2, and 1.2–1.5 m of the combined full-field (20 soil sample locations) and southeast-corner (20 soil sample locations) surveys.

Soil Property ^†^	No. of Sample Sites	Mean	Minimum	Maximum	Range	Standard Deviation	Standard Error	Coefficient of Variation	Skewness	Kurtosis
Depth 0–0.15 m										
θ_g_, kg kg^−1^	40	0.10	0.01	0.27	0.26	0.05	0.01	47.9	1.11	1.86
SP	40	53.41	44.26	65.45	21.19	5.49	0.87	10.3	0.40 ^‡^	−0.81 ^‡^
EC_e_, dS m^−1^	40	10.29	2.54	33.00	30.46	7.74	1.22	75.2	0.92	0.08 ^‡^
pH_e_	40	7.29	6.64	8.45	1.80	0.33	0.05	4.5	1.29	3.29
Cl^−^, meq L^−1^	40	64.07	12.67	204.95	192.28	57.52	9.09	89.8	0.98	−0.36 ^‡^
HCO_3_^−^, meq L^−1^	40	2.91	1.28	7.59	6.31	1.54	0.24	52.8	1.32	1.41 ^‡^
PO_4_^−^, meq L^−1^	40	0.23	0.04	0.70	0.66	0.14	0.02	60.3	1.52	3.18
NO_3_^−^, meq L^−1^	40	4.19	0.69	17.99	17.31	4.35	0.69	103.7	1.32	1.08 ^‡^
SO_4_^2−^, meq L^−1^	40	51.76	7.20	343.94	336.74	61.99	9.80	119.8	3.03	12.12
SAR	40	6.53	2.26	40.33	38.07	7.73	1.22	118.4	2.90	9.39
Na^+^, meq L^−1^	40	10.97	5.21	48.07	42.86	8.20	1.30	74.7	2.80	10.06
K^+^, meq L^−1^	40	62.14	11.58	360.84	349.26	66.69	10.54	107.3	2.57	9.28
Ca^2+^, meq L^−1^	40	1.85	1.00	4.11	3.11	0.70	0.11	37.9	1.91	3.98
Mg^2+^, meq L^−1^	40	19.89	4.42	57.50	53.08	13.96	2.21	70.2	0.80	−0.25 ^‡^
B, mg L^−1^	40	33.93	5.24	105.38	100.14	29.27	4.63	86.3	0.93	−0.35 ^‡^
Depth 0.15–0.3 m										
θ_g_, kg kg^−1^	40	0.13	0.03	0.24	0.21	0.06	0.01	43.1	0.24 ^‡^	−1.29 ^‡^
SP	40	53.86	39.02	63.83	24.81	6.47	1.02	12.0	−0.21 ^‡^	−0.67 ^‡^
EC_e_, dS m^−1^	40	9.50	2.01	33.70	31.69	7.73	1.22	81.4	1.14	0.93 ^‡^
pH_e_	40	7.34	6.70	8.62	1.92	0.32	0.05	4.4	1.54	5.24
Cl^−^, meq L^−1^	40	42.42	8.04	132.50	124.46	39.31	6.22	92.7	1.03	−0.44 ^‡^
HCO_3_^−^, meq L^−1^	40	2.33	0.78	5.01	4.24	1.12	0.18	48.2	0.84	−0.26 ^‡^
PO_4_^−^, meq L^−1^	40	0.20	0.00	0.93	0.93	0.15	0.02	78.2	2.93	12.69
NO_3_^−^, meq L^−1^	40	2.24	0.58	6.26	5.68	1.65	0.26	73.7	0.86	−0.51 ^‡^
SO_4_^2−^, meq L^−1^	40	71.28	6.93	370.34	363.41	78.73	12.45	110.5	1.89	4.25
SAR	40	10.71	1.99	58.32	56.34	15.06	2.38	140.6	2.33	4.60
Na^+^, meq L^−1^	40	12.58	5.15	48.78	43.63	9.73	1.54	77.3	2.20	5.03
K^+^, meq L^−1^	40	66.41	10.03	360.01	349.98	72.63	11.48	109.4	2.12	5.73
Ca^2+^, meq L^−1^	40	1.20	0.43	3.80	3.37	0.56	0.09	46.8	2.80	11.32
Mg^2+^, meq L^−1^	40	15.63	3.02	36.93	33.91	10.64	1.68	68.1	0.41 ^‡^	−1.23 ^‡^
B, mg L^−1^	40	30.09	3.69	89.71	86.01	27.12	4.29	90.1	0.84	−0.70 ^‡^
Depth 0.3–0.6 m										
θ_g_, kg kg^−1^	40	0.18	0.12	0.25	0.13	0.04	0.01	23.4	0.04 ^‡^	−1.34 ^‡^
SP	40	54.85	44.90	63.64	18.74	4.99	0.79	9.1	−0.20 ^‡^	−0.77 ^‡^
EC_e_, dS m^−1^	40	12.64	1.28	47.00	45.72	11.49	1.82	90.9	1.45	1.69
pH_e_	40	7.53	6.32	8.76	2.44	0.42	0.07	5.6	−0.10 ^‡^	1.97
Cl^−^, meq L^−1^	40	52.37	4.44	186.89	182.46	55.67	8.80	106.3	1.20	0.11 ^‡^
HCO_3_^−^, meq L^−1^	40	1.71	0.67	3.83	3.16	0.58	0.09	33.8	1.66	4.61
PO_4_^−^, meq L^−1^	40	0.18	0.00	0.74	0.74	0.21	0.03	117.7	1.35	1.08 ^‡^
NO_3_^−^, meq L^−1^	40	1.33	0.52	4.60	4.08	0.83	0.13	62.2	1.93	5.06
SO_4_^2−^, meq L^−1^	40	121.15	4.58	643.29	638.71	140.95	22.29	116.3	2.10	4.59
SAR	40	19.04	1.83	103.37	101.54	25.51	4.03	133.9	2.13	3.85
Na^+^, meq L^−1^	40	17.59	5.69	69.43	63.74	15.58	2.46	88.6	2.26	4.66
K^+^, meq L^−1^	40	108.07	7.28	598.83	591.55	133.51	21.11	123.5	2.16	4.59
Ca^2+^, meq L^−1^	40	0.91	0.18	3.50	3.32	0.77	0.12	84.7	1.70	2.66
Mg^2+^, meq L^−1^	40	17.35	1.47	44.45	42.98	11.72	1.85	67.6	0.15 ^‡^	−0.93 ^‡^
B, mg L^−1^	40	41.29	1.80	141.13	139.33	38.29	6.05	92.7	1.08	0.64 ^‡^
Depth 0.6–0.9 m										
θ_g_, kg kg^−1^	40	0.21	0.17	0.26	0.09	0.03	0.004	12.9	0.14 ^‡^	−1.38 ^‡^
SP	40	54.14	46.03	70.77	24.74	5.80	0.92	10.7	0.72 ^‡^	0.37 ^‡^
EC_e_, dS m^−1^	40	12.49	1.57	46.00	44.43	11.21	1.77	89.8	1.36	1.27 ^‡^
pH_e_	40	7.74	6.80	8.93	2.13	0.44	0.07	5.6	−0.13 ^‡^	0.84 ^‡^
Cl^−^, meq L^−1^	40	49.10	1.67	205.33	203.67	51.18	8.09	104.2	1.35	1.05 ^‡^
HCO_3_^−^, meq L^−1^	39	1.85	0.80	3.90	3.10	0.60	0.10	32.7	1.34	2.22
PO_4_^−^, meq L^−1^	40	0.15	0.00	0.56	0.56	0.17	0.03	116.3	1.09	0.12 ^‡^
NO_3_^−^, meq L^−1^	40	0.66	0.20	1.74	1.54	0.34	0.05	52.0	1.53	2.38
SO_4_^2−^, meq L^−1^	40	117.17	7.11	504.59	497.48	135.06	21.35	115.3	1.75	2.32
SAR	40	18.94	1.99	80.32	78.33	22.26	3.52	117.5	1.76	2.10
Na^+^, meq L^−1^	40	18.98	7.23	61.72	54.48	14.74	2.33	77.6	1.91	2.74
K^+^, meq L^−1^	40	108.10	10.43	450.35	439.92	125.52	19.85	116.1	1.75	2.16
Ca^2+^, meq L^−1^	40	0.81	0.14	3.15	3.01	0.75	0.12	92.0	1.57	2.05
Mg^2+^, meq L^−1^	40	15.36	1.80	35.49	33.68	10.85	1.72	70.6	0.09 ^‡^	−1.64
B, mg L^−1^	40	36.89	2.36	152.45	150.09	38.00	6.01	103.0	1.38	1.59
Depth 0.9–1.2 m										
θ_g_, kg kg^−1^	40	0.22	0.18	0.26	0.08	0.02	0.003	9.1	0.43 ^‡^	−0.74 ^‡^
SP	40	51.21	39.47	75.44	35.96	8.80	1.39	17.2	1.03	0.41 ^‡^
EC_e_, dS m^−1^	40	9.78	2.24	41.50	39.26	9.07	1.43	92.8	1.66	2.79
pH_e_	40	7.90	7.10	8.30	1.21	0.31	0.05	3.9	−0.72 ^‡^	−0.31 ^‡^
Cl^−^, meq L^−1^	40	33.06	4.44	163.47	159.03	33.83	5.35	102.3	2.07	5.24
HCO_3_^−^, meq L^−1^	40	2.15	1.10	3.88	2.77	0.78	0.12	36.3	0.48 ^‡^	−1.15 ^‡^
PO_4_^−^, meq L^−1^	40	0.10	0.00	0.39	0.39	0.12	0.02	113.3	1.03	0.00 ^‡^
NO_3_^−^, meq L^−1^	39	0.46	0.15	1.30	1.16	0.26	0.04	55.5	1.82	3.72
SO_4_^2−^, meq L^−1^	40	90.62	10.68	418.43	407.76	102.12	16.15	112.7	1.68	2.37
SAR	40	14.18	2.20	66.34	64.14	17.39	2.75	122.6	1.68	1.75
Na^+^, meq L^−1^	40	16.75	5.00	54.08	49.08	12.61	1.99	75.3	1.82	2.27
K^+^, meq L^−1^	40	80.93	14.28	389.51	375.22	94.43	14.93	116.7	1.85	2.82
Ca^2+^, meq L^−1^	40	0.60	0.12	2.12	2.00	0.51	0.08	85.4	1.39	1.31 ^‡^
Mg^2+^, meq L^−1^	40	13.70	2.02	28.78	26.76	10.53	1.66	76.9	0.10 ^‡^	−1.89
B, mg L^−1^	40	24.95	3.28	118.77	115.49	26.92	4.26	107.9	1.99	4.60
Depth 1.2–1.5 m										
θ_g_, kg kg^−1^	40	0.24	0.21	0.30	0.09	0.02	0.004	9.8	0.61 ^‡^	0.03 ^‡^
SP	40	48.13	36.59	67.57	30.98	8.61	1.36	17.9	0.85	−0.35 ^‡^
EC_e_, dS m^−1^	40	7.91	2.09	33.80	31.71	7.24	1.15	91.6	1.75	3.17
pH_e_	40	7.98	7.39	8.34	0.95	0.27	0.04	3.4	−0.68 ^‡^	−0.67 ^‡^
Cl^−^, meq L^−1^	40	22.92	3.39	117.63	114.23	24.12	3.81	105.2	2.55	7.36
HCO_3_^−^, meq L^−1^	40	2.18	0.97	3.86	2.88	0.75	0.12	34.6	0.47 ^‡^	−0.80 ^‡^
PO_4_^−^, meq L^−1^	40	0.09	0.00	0.39	0.39	0.11	0.02	128.0	1.31	0.96 ^‡^
NO_3_^−^, meq L^−1^	40	0.36	0.14	1.04	0.90	0.21	0.03	56.7	1.65	2.78
SO_4_^2−^, meq L^−1^	40	72.13	9.72	336.59	326.88	78.87	12.47	109.3	1.56	2.11
SAR	40	10.72	1.72	50.15	48.43	13.78	2.18	128.5	1.69	1.69
Na^+^, meq L^−1^	40	14.55	4.28	45.79	41.51	10.28	1.63	70.7	1.78	2.16
K^+^, meq L^−1^	40	62.22	14.00	328.97	314.97	72.11	11.40	115.9	2.01	4.04
Ca^2+^, meq L^−1^	40	0.49	0.10	1.71	1.61	0.43	0.07	87.1	1.29	0.83 ^‡^
Mg^2+^, meq L^−1^	40	11.76	1.49	25.69	24.20	9.67	1.53	82.3	0.29 ^‡^	−1.88
B, mg L^−1^	40	18.26	2.93	81.23	78.31	18.69	2.95	102.3	1.95	4.33

^†^ Definitions: θ_g_ = gravimetric water content, SP = saturation percentage, EC_e_ = electrical conductivity of the saturation extract, SAR = sodium adsorption ratio. ^‡^ Significant. Skewness is significant if skewness divided by standard error of skewness > 2. Kurtosis is significant if kurtosis divided by stand error of kurtosis > 2.

**Table 2:** Soil edaphic property mean and range statistics of the composite depths of (a) 0–1.5 m and (b) 0–1.2 m for the combined full-field (20 soil sample locations) and southeast-corner (20 soil sample locations) surveys.

Soil Property ^†^	No. of Sample Sites	Mean	Min.	Max.	Range	Standard Deviation	Standard Error	Coefficient of Variation	Skewness	Kurtosis
(a) Depth 0–1.5 m										
θ_g_, kg kg^−1^	40	0.18	0.14	0.24	0.10	0.03	0.005	16.1	0.26 ^‡^	−1.17 ^‡^
SP	40	52.97	43.46	64.56	21.10	4.93	0.78	9.3	0.10 ^‡^	−0.57 ^‡^
EC_e_, dS m^−1^	40	10.70	2.05	36.22	34.17	9.03	1.43	84.4	1.30	1.13 ^‡^
pH_e_	40	7.63	7.18	8.53	1.35	0.27	0.04	3.6	0.68 ^‡^	1.80
Cl^−^, meq L^−1^	40	44.09	8.16	152.11	143.95	40.08	6.34	90.9	1.08	0.08 ^‡^
HCO_3_^−^, meq L^−1^	40	2.12	1.18	3.40	2.22	0.60	0.09	28.4	0.30 ^‡^	−0.72 ^‡^
PO_4_^−^, meq L^−1^	40	0.16	0.01	0.58	0.57	0.14	0.02	91.5	1.27	1.03 ^‡^
NO_3_^−^, meq L^−1^	40	1.40	0.48	4.52	4.04	0.96	0.15	68.8	1.22	1.18 ^‡^
SO_4_^2−^, meq L^−1^	40	92.43	7.95	420.42	412.47	101.32	16.02	109.6	1.75	2.63
SAR	40	31.86	3.48	112.13	108.66	28.73	4.54	90.2	1.10	0.74 ^‡^
Na^+^, meq L^−1^	40	14.30	2.17	65.31	63.14	17.76	2.81	124.2	1.91	2.59
K^+^, meq L^−1^	40	15.73	6.86	54.73	47.86	12.08	1.91	76.8	2.04	3.37
Ca^2+^, meq L^−1^	40	85.14	11.56	398.95	387.39	95.56	15.11	112.2	1.85	2.88
Mg^2+^, meq L^−1^	40	0.94	0.32	3.00	2.68	0.58	0.09	61.8	1.83	3.66
B, mg L^−1^	40	15.57	2.65	35.06	32.41	10.45	1.65	67.1	0.15 ^‡^	−1.63
(b) Depth 0–1.2 m										
θ_g_, kg kg^−1^	34 ^£^	0.19	0.12	0.25	0.13	0.05	0.01	24.9	−0.12 ^‡^	−1.60 ^‡^
SP	34 ^£^	53.23	45.83	61.61	15.77	4.01	0.69	7.5	0.29 ^‡^	−0.54 ^‡^
EC_e_, dS m^−1^	34 ^£^	9.97	1.84	29.97	28.13	6.29	1.08	63.0	0.99	1.57 ^‡^
pH_e_	34 ^£^	7.74	7.06	8.74	1.68	0.33	0.06	4.2	0.46 ^‡^	1.50 ^‡^
Cl^−^, mEq L^−1^	34 ^£^	51.83	7.87	279.17	271.30	54.37	9.32	104.9	2.53	8.49
PO_4_^−^, mEq L^−1^	34 ^£^	0.19	0.00	1.11	1.11	0.24	0.04	126.4	2.01	4.89
SO_4_^2−^, mEq L^−1^	34 ^£^	67.16	6.40	165.86	159.46	41.82	7.17	62.3	0.41 ^‡^	−0.36 ^‡^
SAR	34 ^£^	12.06	6.08	22.47	16.40	4.02	0.69	33.4	0.71 ^‡^	0.12 ^‡^
Na^+^, mEq L^−1^	34 ^£^	63.68	9.91	166.12	156.21	41.48	7.11	65.1	0.71 ^‡^	0.00 ^‡^
K^+^, mEq L^−1^	34 ^£^	0.92	0.39	3.91	3.51	0.60	0.10	65.1	4.05	19.62
Ca^2+^, mEq L^−1^	34 ^£^	17.65	2.68	53.90	51.22	11.34	1.95	64.3	0.87	1.64 ^‡^
Mg^2+^, mEq L^−1^	34 ^£^	33.94	3.23	112.97	109.75	25.44	4.36	75.0	0.95	1.27 ^‡^
B, mg L^−1^	34 ^£^	10.03	2.14	24.24	22.10	6.06	1.04	60.4	0.60 ^‡^	−0.59 ^‡^
LF ^§^	34 ^£^	0.27	0.08	0.61	0.53	0.15	0.02	53.1	0.61 ^‡^	−0.30

^†^ Definitions: θ_g_ = gravimetric water content, SP = saturation percentage, EC_e_ = electrical conductivity of the saturation extract, SAR = sodium adsorption ratio, LF = leaching fraction. ^‡^ Significant. Skewness is significant if skewness divided by standard error of skewness > 2. Kurtosis is significant if kurtosis divided by stand error of kurtosis > 2. ^§^ Leaching fraction was determined by dividing the Cl^-^ concentration of the irrigation water by the Cl^−^ concentration of the saturation extract at the 1.2–1.5 m depth increment at each site where the oilseed yield was greater than zero. ^£^ Sites where oilseed yield was greater than zero. These sites were used for Ida Gold mustard oilseed yield model development.

**Table 3:** Correlation coefficients between edaphic properties and both EC_a_ and oilseed yield that are significantly correlated. ^†^

Edaphic Property ^‡^	EC_a_	Oilseed Yield
θ_g_	0.73 **	0.46 **
EC_e_	0.98 **	−0.41 **
Boron	0.88 **	−0.32 *
pH_e_	−0.09	0.28
SAR	0.87 **	−0.30
LF	0.80 **	0.55 **
SP	0.45 **	−0.45 **

**^†^** Using 34 locations where yield > 0. ^‡^ Averaged over 0–1.2 m. Definitions: θ_g_ = gravimetric water content, SP = saturation percentage, EC_e_ = electrical conductivity of the saturation extract, SAR = sodium adsorption ratio. * Significant at *p* < 0.05 level. ** Significant at *p* < 0.01 level.

**Table 4:** Degree of predicted oilseed yield sensitivity to 1 standard deviation (SD) change in each edaphic property of Equation (3). ^†^

Parameter Sensitivity ^‡^	Calculated Yield (kg ha^−1^)	Conversion to Biofuel (L ha^−1^)	Percentage Change (%)	Boron (mg L^−1^)	EC_e_ (dS m^−1^)	LF	θ_g_ (kg kg^−1^)
Baseline	1017.3	178.3		4.0	6.8	0.27	0.19
B + 1 SD	345.2	60.5	−66.1	10.1	6.8	0.27	0.19
EC_e_ + 1 SD	776.2	136.0	−23.7	4.0	13.1	0.27	0.19
LF + 1 SD	1212.3	212.5	19.2	4.0	6.8	0.42	0.19
θ_g_ + 1 SD	1033.3	181.1	1.6	4.0	6.8	0.27	0.24

^†^ For the 0–1.2 m composite depth increment. ^‡^ Definitions: B = boron, EC_e_ = electrical conductivity of the saturation extract, LF = leaching fraction, θ_g_ = gravimetric water content.

**Table 5:** Summary of the average residual, standard deviation of the residual, and data count for each category of predicted salinity (i.e., EC_e_) from Scudiero et al. [29], indicating the uncertainty of the EC_e_ prediction and used in defining the EC_e_ residual PDFs.

Lower Limit of EC_e_ Interval (dS m^−1^)	Average EC_e_ Residual (dS m^−1^)	Standard Deviation of the EC_e_ Residuals (dS m^−1^)	Data Count
0	−2.29	3.38	131
1	−0.38	2.13	577
2	−0.68	2.39	623
3	−0.32	2.21	584
4	−0.86	2.62	351
5	−0.97	2.45	245
6	−0.95	2.49	298
7	−0.75	2.69	267
8	−0.86	3.01	215
9	−0.52	2.67	143
10	−0.50	3.03	82
11	1.27	2.38	83
12	2.18	1.51	121
13	3.00	1.69	193
14	3.64	1.65	127
15	4.91	2.07	77
16	6.57	2.60	194
Entire data set	0.14	3.11	4311

Definition: EC_e_ = electrical conductivity of the saturation extract.

## Abbreviations

The following abbreviations are used in this manuscript:

EC_e_	Electrical Conductivity of the Saturation Extract (dS m^−1^)
EM_h_, EC_a_	Measured with Electromagnetic Induction in the Horizontal Coil Configuration (dS m^−1^)
EM_v_, EC_a_	Measured with Electromagnetic Induction in the Vertical Coil Configuration (dS m^−1^)
EMI	Electromagnetic Induction
SJV	San Joaquin Valley
WSJV	West Side of the San Joaquin Valley
